# The Effect of Theobromine Supplementation on HDL‐c Subclasses (HDL‐c2, HDL‐c3), HDL‐c2/HDL‐c3 Ratio, and Gene Expression of PPAR‐α and Sirt1 in Subjects With Metabolic Syndrome: A Randomized Controlled Clinical Trial

**DOI:** 10.1002/fsn3.70665

**Published:** 2025-07-16

**Authors:** Elham Sharifi‐Zahabi, Zahra Dastafkan, Amirhossein Asadi, Fatemeh Sadat Hosseini‐Baharanchi, Nayebali Rezvani, Saba Yari, Farzad Shidfar

**Affiliations:** ^1^ Department of Nutrition, School of Public Health Iran University of Medical Sciences Tehran Iran; ^2^ Kermanshah University of Medical Sciences Kermanshah Iran; ^3^ Medical Genetics Laboratory Kermanshah University of Medical Sciences Kermanshah Iran; ^4^ Student Research Committee, School of Nutrition and Food Sciences Shiraz University of Medical Sciences Shiraz Iran; ^5^ Minimally Invasive Surgery Research Center, Department of Biostatistics, School of Public Health Iran University of Medical Sciences Tehran Iran; ^6^ Department of Clinical Biochemistry Kermanshah University of Medical Sciences Kermanshah Iran; ^7^ Nutritional Sciences Research Center Iran University of Medical Sciences Tehran Iran

**Keywords:** HDL‐c, metabolic syndrome, PPAR alpha, theobromine

## Abstract

Several studies have reported the beneficial effects of theobromine (TB) on metabolic syndrome (MetS) indices and cardiovascular risk factors. However, the findings are controversial. The current study aimed to examine the effects of a 12‐week intake of pure TB in combination with a low‐calorie diet on serum levels of HDL‐c2, HDL‐c3, the HDL‐c2/HDL‐c3 ratio, and the gene expression of peroxisome proliferator‐activated receptor alpha (PPAR‐α) and silent information regulator 1 (Sirt1) in overweight and obese adults with MetS. In this randomized clinical trial, 80 participants with MetS were randomly allocated to intake a 450 mg/day TB or placebo in combination with a low‐calorie diet for 12 weeks. Dietary intake, anthropometric indices, fasting serum levels of HDL‐c2 and HDL‐c3, the HDL‐c2/HDL‐c3 ratio, and the gene expression of PPAR‐α and Sirt1 were assessed at the start and end of the study. The sample size at completion was *n* = 72 subjects. Our findings revealed that TB supplementation significantly increased HDL‐c (0.34 ± 2.11 vs. −1.24 ± 3.09; *p* = 0.041), HDL‐c2 (0.95 ± 1.8 vs. −0.26 ± 1.30; *p* < 0.001), the HDL‐c2/HDL‐c3 ratio (0.04 ± 0.45 vs. 0.01 ± 0.04; *p* = 0.004), and the gene expression of PPAR‐α (0.884 ± 0.195 vs. 0.332 ± 0.178; *p* = 0.004) compared to the placebo. The results of the current study showed that TB supplementation led to an increase in serum levels of HDL‐c2, the HDL‐c2/HDL‐c3 ratio, and the gene expression of PPAR‐α in subjects with MetS.

**Trial Registration:** IRCT20091114002709N59

## Graphical abstract

The results of the current study showed that theobromine supplementation led to an increase in serum levels of HDL‐c2, HDL‐c2/HDL‐c3, and gene expression of PPAR‐α in subjects with MetS.

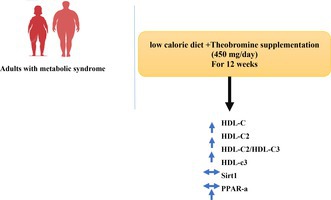

**Abbreviations**
ABCA1: adenosine triphosphate‐binding cassette transporter A1
ApoA1: apolipoprotein A1
ApoB: apolipoprotein B
BMI: body mass index
CETP: cholesteryl ester transfer protein
CVD: cardiovascular diseases
DBP: diastolic blood pressure
HDL‐c: high‐density cholesterol
LDL‐c: low‐density cholesterol
MET: metabolic equivalent of task
NAD: nicotinamide adenine dinucleotide
PA: physical activity
PPAR‐α: peroxisome proliferator‐activated receptor α
SBP: systolic blood pressure
TB: theobromine
TG: triglyceride
WC: waist circumference
WHR: waist‐to‐hip ratio

## 1 Introduction

The prevalence of metabolic syndrome (MetS), manifested by hypertriglyceridemia, low high‐density lipoprotein cholesterol (HDL‐c), high blood glucose, abdominal obesity, and high blood pressure, keeps increasing worldwide. A clear link between MetS and increased risk of cardiovascular diseases (CVDs) and type 2 diabetes has been shown by several studies (Jialal et al. 2015; Ahima 2024). Lifestyle modifications are among the most common interventional strategies for managing MetS. Besides, as the disease severity worsens, statins and antihypertensive drugs are considered along with the ongoing lifestyle modifications. However, interventions aiming at optimizing dietary intakes may attenuate the risk of MetS in subjects with no medication and serve as an adjunct to those who receive standard pharmacologic therapies (Abbate et al. 2020). In recent years, the administration of dietary supplements to reduce CVDs risk factors has attracted a great deal of attention. In this regard, a special focus has been made on the role of theobromine (TB), the main methyl‐xanthine in cocoa (Berends et al. 2015). The beneficial effects of TB on fasting blood lipids and certain MetS risk factors have been demonstrated in several studies (Smolders et al. 2018; Neufingerl et al. 2013). In a study, Neufingerl et al. (2013) revealed that the administration of 850 mg/day of TB led to an increase in serum HDL‐c concentrations in healthy subjects. In another study, a daily intake of 500 mg TB could decrease low‐density lipoprotein cholesterol (LDL‐c) in subjects with low baseline HDL‐c, while no significant effects on HDL‐c, vascular function, insulin, and blood glucose were observed (Smolders et al. 2018). Likewise, in a study on subjects with MetS, we showed that TB supplementation along with a low‐calorie diet could increase HDL‐c and reduce LDL‐c/HDL‐c ratio, triglyceride to HDL‐c ratio (TG/HDL‐c), and total cholesterol to HDL‐c ratio (TC/HDL‐c) (Sharifi‐Zahabi et al. 2023). Low HDL‐c levels typically suggest a higher risk of cardiovascular disease, especially in primary prevention (März et al. 2017). In a post hoc analysis of the TNT (Treating to New Targets) trial, Barter et al. (2007) reported that HDL‐c levels were a notable negative predictor of future major cardiovascular events. Considering an inverse association between HDL‐c and CVDs reported by epidemiological studies, increasing HDL‐c has been suggested as a potential therapeutic target (Cho et al. 2020). However, the plasma level of HDL‐c may not reflect the function of HDL‐c, as the change in HDL‐c composition can cause HDL‐c biological dysfunction (Pirillo et al. 2019). Indeed, this lipoprotein can be classified into two main sub‐particles, HDL‐c2 and HDL‐c3, based on their densities following ultracentrifugation (Soran et al. 2012). Previous studies have shown an inverse association of HDL‐c2 and HDL‐c2/HDL‐c3 ratio with MetS and CVD (Moriyama et al. 2014; Hwang et al. 2019). In a study conducted on heart failure patients, a reduced level of cholesterol in circulating HDL‐c3 particles was linked to a higher 3‐month mortality (Degoricija et al. 2019). Additionally, another study reported an inverse association between denser HDL‐c3 levels and the incidence of coronary heart disease (Joshi et al. 2016). With an increase in MetS components, the HDL phenotype encompasses a higher amount of small HDL‐c3 and fewer large HDL‐c2, leading to a lower HDL‐c2/HDL‐c3 ratio (Moriyama et al. 2014). Taken together, this background indicates that assessing only HDL‐c is not enough to understand the effect of dietary interventions on its functionality and antiatherogenic role.

Although the precise mechanism for the effects of TB on the lipid profile has not yet been completely understood, suggested mechanisms include increased fatty acid β‐oxidation and decreased hepatic de novo lipogenesis. In mouse hepatocytes, TB induced lipid metabolism by upregulating peroxisome proliferator‐activated receptor alpha (PPAR‐α) and carnitine palmitoyl transferase 1α (CPT1‐α), while simultaneously inhibiting sterol regulatory element binding protein 1‐c (SREBP1‐c) (Wei et al. 2021). Moreover, recent research has shown that TB has antidiabetic effects by decreasing oxidative stress in the kidneys of diabetic rats through the activation of silent information regulator 1 (Sirt1), a nicotinamide adenine dinucleotide (NAD)‐dependent histone deacetylase (Papadimitriou et al. 2015). Sirt1 regulates various cellular processes, including glucose metabolism, fatty acid beta oxidation, and stress response (Kosgei et al. 2020). Both Sirt1 and PPAR‐α are important regulators of lipid metabolism, and their interaction plays a pivotal role in metabolic homeostasis (Rkhaya et al. 2018; Lin et al. 2022). Sirt1 can deacetylate PPAR‐α, thereby increasing its transcriptional activity. This interaction enhances the expression of genes involved in fatty acid oxidation, such as acyl‐CoA oxidase and CPT1 (Kosgei et al. 2020; Todisco et al. 2022). Hence, it is thought that the favorable effect of TB on lipid metabolism may be mediated by an increase in the activity of Sirt1 and PPAR‐α (Wei et al. 2021; Papadimitriou et al. 2015). As previously noted, in our previous work (Sharifi‐Zahabi et al. 2023), we showed a significant increase in HDL‐c following 12 weeks of TB intake (450 mg/day). Considering that clinical trials in the field of HDL‐c subfractions are limited, the present study was designed to find out which HDL‐c subfraction accounts for the observed increase in HDL‐c. Additionally, we aimed to assess the effect of TB on the HDL‐c2/HDL‐c3 ratio and the expression of genes involved in the lipid metabolism pathway (PPAR‐α and Sirt1) in overweight and obese adults with MetS. The hypothesis of the study was that TB has an increasing effect on HDL‐c via increasing the expression of both Sirt1 and PPAR‐α genes.

## 2 Materials and Methods

### 2.1 Participants

The current study was performed at the comprehensive health centers of Kermanshah University of Medical Sciences, Kermanshah, Iran, between January and September 2022. Overweight and obese individuals (BMI 25–35 kg/m^2^) aged 40–55 years, with MetS were invited to participate in this study. Inclusion criteria based on the International Diabetes Federation (IDF) criteria (Alberti et al. 2005) were: a waist circumference (WC) > 94 cm in men and > 80 cm in women plus any two of the following: TG > 150 mg/dL or specific treatment for this lipid abnormality, HDL‐c < 40 mg/dL in men and < 50 mg/dL in women or specific treatment for this condition, systolic blood pressure (SBP) ≥ 130 or diastolic blood pressure (DBP) ≥ 85 mmHg or treatment of previously diagnosed hypertension, and fasting blood glucose (FBG) ≥ 100 mg/dL. Likewise, exclusion criteria were: ≤ 2 MetS components, smoking history, any known chronic diseases such as diabetes, vascular disease, uncontrolled hypertension, cancer, heart, gastrointestinal, liver, lung, and kidney diseases, change in medication dosage, intake of dietary supplements, hypoglycemic, vasodilator, and hormone‐replacement medications. Those medicated with antihypertensive or statin (or a combination) drugs were included if they were habituated (treatment with antihypertension medications for more than 6 months, and statins for more than 3 months) (Curtis et al. 2019). The ethics committee of Iran University of Medical Sciences, Tehran, Iran (IR.IUMS.REC.1400.761) approved the study protocol. The registration number for the study was IRCT20091114002709N59.

### 2.2 Study Procedure

The present study was a 12‐week double‐blinded, randomized controlled trial. The full description of the study protocol has been provided in our previous article (Sharifi‐Zahabi et al. 2023). Briefly, all eligible participants received a low‐calorie (300–500 kcal/day deficit) diet and then randomly assigned to TB or placebo (maltodextrin) group using a stratified block randomization design with a block size of four matched for BMI (25–30 and 30–35 kg/m^2^) (Sharifi‐Zahabi et al. 2023). Subjects in each stratum were assigned to the treatment or placebo group based on a random list generated by PASS 11 statistical software. The random list contained 3‐digit codes for each participant that indicated the corresponding treatment. The codes were provided to the researcher and labeled on the drug bottles. To maintain allocation concealment of the study, only the study project executive expert was aware of the designed codes, while the investigators, those who assessed outcomes and participants, were blinded.

The daily calorie requirement was determined based on the equation recommended by the Institute of Medicine, Food and Nutrition Board, considering participants' age, sex, weight, height, and physical activity (PA) level (Trumbo et al. 2002). The TB powder was purchased from Bulk Supplements, USA, and the maltodextrin powder was provided by Karen Pharmaceuticals and Vital Food Supplements Company, Tehran, Iran. Both TB and maltodextrin powder were capsulated at the School of Pharmacy of Shahid Beheshti University of Medical Sciences, Tehran, Iran. Participants in the TB group received a capsule containing 450 mg TB and 50 mg maltodextrin, while those in the placebo group received the same amount of maltodextrin capsule, with a similar size and shape, for 12 weeks. A list of cocoa‐containing foods was provided, and all participants were requested to avoid these products before and during the study. Moreover, as the body's caffeine is metabolized to TB, the intake of caffeine‐containing drinks such as coffee, cola, and tea was limited to a maximum of four cups a day. Participants were requested not to change their daily physical activity level during the study. Furthermore, any adverse events were assessed by asking the subjects or noted by the researchers at each visit. The aim of the study was explained to all subjects, and written informed consent was obtained from each participant before starting the study. The adherence to the dietary advice and supplement intake was evaluated based on each participant's attendance at the periodic visits, assessing the 3‐day food record, and counting the number of unused capsules.

### 2.3 Assessment of Dietary Intake and Physical Activity

A 3‐day food record to evaluate the dietary intakes of participants was collected at baseline, Weeks 4, 6, 8, and 12. Nutritionist IV software was used to analyze the energy and nutrient content. The PA levels were evaluated using the short form of the International PA Questionnaire (IPAQ) at baseline and during the intervention period. The level of PA was calculated as continuous quantitative data, recorded in metabolic equivalent of task (MET)‐min/week, based on the relevant activity coefficients, the frequency of activities during weekdays, and the time (in minutes) spent on each activity. One MET represents the energy consumption during rest and is equal to 3.5 mL O_2_/kg/min. The MET can be determined by weighing each type of activity by its energy requirements. METs are multiples of the resting metabolic rate, and a MET‐minute is calculated by multiplying the MET score of an activity by the number of minutes performed (Committee IPAQ 2003).

The validity and reliability of the PA questionnaire and a 3‐day food record have previously been established (Moghaddam et al. 2012; Yang et al. 2010).

### 2.4 Biochemistry

Venous blood samples were obtained from each participant at the beginning and end of the study, after an overnight fast for 10–12 h. Serum was separated immediately after centrifuging at 3000 × *g* for 10 min. The method for determining fasting HDL‐c has been described in our previous work (Sharifi‐Zahabi et al. 2023). The level of HDL‐c3 was determined according to the one‐step sedimentation method proposed by (Hirano et al. 2008) . In this method, heparin/Mn/DS (heparin–manganese–dextran sulfate) reagents were added to the serum to simultaneously precipitate both the apoB‐containing lipoproteins and HDL‐c2. An aliquot of the supernatant was separated for measurement of HDL‐c3, and the level of HDL‐c3 from the supernatant was determined using a direct enzymatic method by the Delta treatment kit (AUDIT, Tehran, Iran). To determine HDL‐c2 levels, HDL‐c3 values were subtracted from total HDL‐c values.

### 2.5 Gene Expression of PPAR‐α and Sirt 1

Buffy coat of white blood cells was separated following centrifugation of blood samples. Then, ribonucleic acid (RNA) was extracted via the Stem Cell Research Center kit (Trisol, ytizol Pure RNA, CAT:yT9064, Iran). After that, complementary DNA (cDNA) was synthesized by Parstous kit (Easy cDNA Synthesis; Lot:752521, Iran) and was used for Real‐time polymerase chain reaction (PCR) based on the protocols provided by Aryan Gene Gostar kit (qPCRBIO SYGreen Mix Hi‐R‐100 × 20 μL rxns, Iran). The GAPDH (glyceraldehyde‐3‐phosphate dehydrogenase) was the housekeeping gene used in real‐time PCR. The primer sequences that were used in real‐time PCR are presented in Table 1.

**TABLE 1:** Primers used in the current study.

Genes	Sequence
GAPDH forward primer (1)	ACAACTTTGGTATCGTGGAAGG
GAPDH reverse primer (2)	GCCATCACGCCACAGTTTC
Sirt1 forward primer (1)	TGTGCTTGTGGACTCTCACAT
Sirt1 reverse primer (2)	GGATTCCCAGTGCGAGTAGAT
PPAR‐α forward primer (1)	ATG GTG GAC ACG GAA AGC C
PPAR‐α reverse primer (2)	CGATGGATTGCGAAATCTCTTGG

### 2.6 Statistical Analysis

Statistical analysis was conducted using IBM SPSS software version 22 (SPSS Inc.). The formula for parallel clinical trials, *N* = [(*Z*_1 − *α*/2_ + *Z*_1 − *β*_)^2^ × (*S*_1_^2^ + *S*_2_^2^)]/(Δ)^2^, and HDL‐c as the principal variable (Neufingerl et al. 2013), were used for calculating the study sample size. Considering a type I error of 5% (*α* = 0.05), type II error of 20% (*β* = 0.20, power = 80%), *S*_1_ = 0.49, *S*_2_ = 0.42, and Δ = 0.3 (based on expert opinion), the minimum sample size was calculated to be 36 per group. Considering a dropout rate of 10%, this was increased to 40 persons for each group. The statistical analysis was performed based on a per‐protocol analysis on all participants who completed the 12‐week intervention (*n* = 72) and an intention‐to‐treat analysis using the mean of variables for missing values on all subjects who were included (*n* = 80) in the study. As the results of both analyses were the same, the per‐protocol results were only reported in this study. The baseline variables between the two groups were compared using the independent samples *t*‐test. Normality was checked based on the Kolmogorov–Smirnov test. The main effects of treatment were assessed by ANCOVA adjusted for confounding variables, including baseline values and changes in BMI and calories. A *p* < 0.05 was considered statistically significant for all the analyses.

## 3 Results

### 3.1 Participants' Characteristics

**FIGURE 1:**
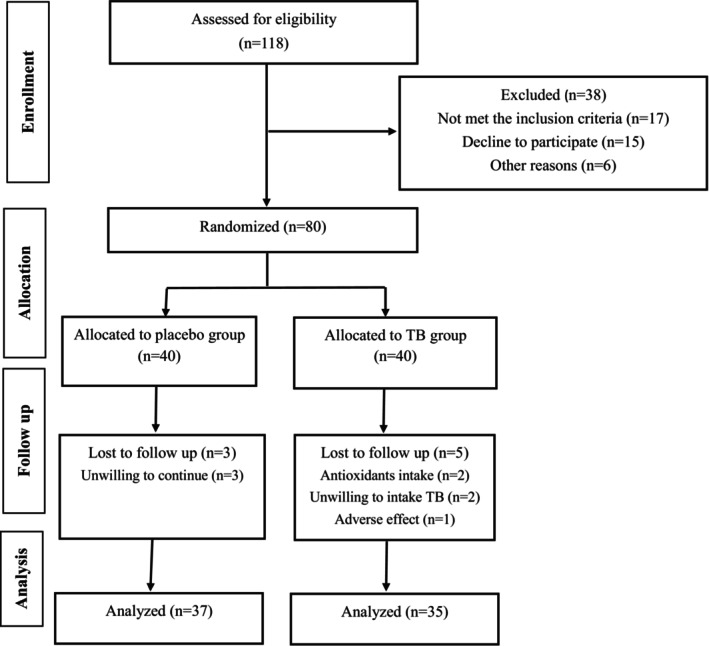
Assignment and progress of participants in the study.

Figure 1 shows the assignment of participants in the study. Initially, 118 individuals volunteered to take part in the study. After assessing for inclusion criteria, 80 subjects met the inclusion criteria and were randomly assigned to receive either TB or a placebo along with a low‐calorie diet for 12 weeks. During the follow‐up, five participants in the TB group and three in the placebo group were excluded due to the reasons mentioned in Figure 1. Hence, a total of 72 subjects (TB, *n* = 35 and placebo, *n* = 37) completed the intervention and were included in the analysis. According to the data in Table 2, the mean age and anthropometric indices of participants were not significantly different between the two groups. Adverse effects were reported in the TB group by five individuals. The most frequently reported side effects were nausea and headache, which resulted in the exclusion of one participant from the intervention. Counting the unused capsules revealed that the compliance of the participants was high, with an average of 90.3% consumption. Table 3 presents the dietary intake and PA levels of participants at the beginning and end of the study. As shown in the table, no significant differences in dietary intake and PA levels were observed between the two groups.

**TABLE 2:** Baseline characteristics of the study participants.

Categorical variables	All (72)		Placebo (*n* = 37)		TB (*n* = 35)		*p* ^a^
	*n*	%	*n*	%	*n*	%	
**Sex**							
Male	7	9.7	3	8.1	4	11.4	0.707
Female	65	90.3	34	91.9	31	88.6	
**Education**							
Elementary	20	27.8	11	29.7	9	25.7	0.838
High school	23	31.9	13	35.1	10	28.6	
Diploma	11	15.3	5	13.5	6	17.1	
Bachelor and higher	18	25.0	8	21.6	10	28.6	
**Drug**							
Antihypertensive drug	21	29.2	8	38.9	13	37.1	0.329
Lipid‐lowering drug	6	8.3	3	8.1	3	8.1	

Continuous variables	Mean	SD	Mean	SD	Mean	SD	*p* ^b^
Age (year)	48.33	4.05	48.18	3.97	48.48	4.19	0.759
Height (cm)	160.73	5.78	1.59	0.05	1.61	0.06	0.253
Body weight (kg)	79.37	9.76	78.01	8.48	80.81	10.89	0.226
BMI (kg/m^2^)	30.66	2.82	30.47	2.83	30.85	2.84	0.570
WC (cm)	102.83	7.21	102.10	6.23	103.60	8.14	0.384
HC (cm)	110.63	5.76	110.21	5.92	111.08	5.63	0.526

Abbreviations: BMI, body mass index; HC, hip circumference; TB, theobromine; WC, waist circumference.

^a^ Based on *χ* ^2^ test.

^b^ Based on independent samples *t*‐test.

**TABLE 3:** Comparison of dietary intakes and physical activity during the study.^a^

Variables	Placebo (*n* = 37)				TB (*n* = 35)				*p*_between_ ^c^
	Baseline	Week 12	Change	*p* ^b^	Baseline	Week 12	Change	*p* ^b^	
Energy (kcal)	2179.48 ± 133.13	1968.20 ± 228.20	−211.27 ± 156.30	< 0.001	2264.85 ± 271.29	2055.0 ± 491.84	−209.45 ± 319.74	< 0.001	0.975
Protein (g)	83.1 ± 5.48	87.9 ± 13.4	4.83 ± 12.4	0.023	86.85 ± 10.40	95.17 ± 23.85	8.32 ± 18.50	0.012	0.349
Carbohydrate (g)	297.17 ± 29.70	266.78 ± 59.18	−30.38 ± 44.4	< 0.001	312.94 ± 49.94	285.60 ± 60.3	−27.33 ± 30.84	< 0.001	0.154
Fat (g)	76.30 ± 6.64	65.85 ± 22.32	−10.44 ± 21.80	0.002	79.80 ± 9.50	69.78 ± 18.30	−10.04 ± 17.80	0.002	0.60
PUFA (g)	18.68 ± 7.57	15.71 ± 7.72	−2.91 ± 0.54	< 0.001	19.94 ± 6.61	17.2 ± 6.70	−2.72 ± 0.4	< 0.001	0.09
MUFA (g)	30.95 ± 3.5	23.12 ± 13.58	−7.83 ± 13.95	0.002	29.8 ± 3.86	25.0 ± 10.77	−4.81 ± 12.17	0.025	0.332
SFA (g)	21.93 ± 6.5	19.40 ± 6.60	−2.50 ± 0.64	< 0.001	20.14 ± 5.70	17.80 ± 5.35	−2.33 ± 1.30	< 0.001	0.482
Fiber (g)	26.55 ± 8.22	25.26 ± 7.72	−1.29 ± 2	< 0.001	31.36 ± 14.31	29.70 ± 13.40	−1.65 ± 2.32	< 0.001	0.480
Caffeine (mg)	50.76 ± 47.76	49.64 ± 48.3	−1.10 ± 6.0	0.266	47.80 ± 42.96	46.60 ± 40.0	−1.2 ± 9.3	0.479	0.975
Physical activity (PA) (MET‐min/week)	874.7 ± 15.32	881.86 ± 16.20	7.16 ± 4.2	0.096	863.48 ± 23.32	870.11 ± 22.7	6.62 ± 4.60	0.159	0.932

Abbreviation: TB, theobromine.

^a^ Values are mean ± SD.

^b^ Based on paired samples *t*‐test.

^c^ Independent samples *t*‐test.

### 3.2 Serum Levels of HDL‐c2, HDL‐c3, and the HDL‐c2/HDL‐c3 Ratio

The serum levels of HDL‐c and its subclasses according to the sex of the population are shown in Table 4. Since the number of male subjects distributed in each group was too small, subgroup analysis based on sex was not performed. According to the data shown in Table 5, there was a significant decrease (−1.24 ± 3.09 and *p* = 0.02) and a nonsignificant increase (+0.34 ± 2.11 and *p* = 0.34) in HDL‐c level after receiving placebo and TB, respectively. The comparison of HDL‐c changes between the two groups showed a significant increase of this lipoprotein in the TB group compared to the placebo group (*p* = 0.041), even after adjusting for confounding variables. Regarding HDL‐c3, although a significant decrease was observed in both groups after the intervention (*p* < 0.05), comparing changes between the two groups did not show a significant difference (*p* = 0.729). Moreover, the serum level of HDL‐c2 showed a significant increase after receiving a low‐calorie diet plus TB (*p* = 0.003), while it decreased nonsignificantly in the placebo group (*p* = 0.23). Comparing the changes between the two groups showed that TB supplementation led to a significant increase in HDL‐c2 levels compared to the placebo, even after adjusting for confounding variables. In the case of the HDL‐c2/HDL‐c3 ratio, a significant increase was observed after TB intake (*p* = 0.001). However, changes in this index were not significant in the placebo group (*p* = 0.673). A comparison of the differences between the two groups showed that, compared to the placebo, TB led to a significant increase in the HDL‐c2/HDL‐c3 ratio (*p* = 0.004).

**TABLE 4:** Serum levels of HDL‐c, HDL‐c2, HDL‐c3, and the HDL‐c2/HDL‐c3 ratio in males and females at baseline.^a^

Variables	Placebo (*n* = 37)		TB (*n* = 35)	
	Male (*n* = 3)	Female (*n* = 34)	Male (*n* = 4)	Female (*n* = 31)
HDL‐c (mg/dL)	38.0 ± 4.60	42.73 ± 6.14	37.50 ± 4.12	41.80 ± 5.02
HDL‐c3	29.60 ± 3.90	32.72 ± 4.23	29.42 ± 2.30	31.50 ± 3.70
HDL‐c2	8.40 ± 1.20	10.01 ± 2.50	8.07 ± 2.05	10.30 ± 2.30
HDL‐c2/HDL‐c3	0.28 ± 0.04	0.30 ± 0.063	0.27 ± 0.05	0.33 ± 0.067

Abbreviations: HDL‐c, high‐density cholesterol; TB, theobromine.

^a^ Values are mean ± SD.

**TABLE 5:** Comparison of serum levels of HDL‐c, HDL‐c2, HDL‐c3, and the HDL‐c2/HDL‐c3 ratio after 12 weeks according to intervention groups.^a^

Variables	Placebo (*n* = 37)				TB (*n* = 35)				Difference^c^	*p*_between_ ^d^	*p*_between_ ^e^
	Baseline	Week 12	Change	*p* ^b^	Baseline	Week 12	Change	*p* ^b^			
HDL‐c (mg/dL)	42.35 ± 6.12	41.10 ± 6.48	−1.24 ± 3.09	0.02	41.28 ± 5.06	41.62 ± 4.14	0.34 ± 2.11	0.34	1.58 ± 3.54	0.014	0.041
HDL‐c3	32.5 ± 4.20	31.5 ± 4.90	−0.98 ± 2.70	0.034	31.25 ± 3.59	30.62 ± 3.04	−0.60 ± 1.7	0.048	0.38 ± 3.1	0.517	0.729
HDL‐c2	9.88 ± 2.50	9.62 ± 2.30	−0.26 ± 1.30	0.23	10.05 ± 3.40	11.00 ± 2.40	0.95 ± 1.8	0.003	1.20 ± 2.10	0.001	< 0.001
HDL‐c2/HDL‐c3	0.30 ± 0.06	0.01 ± 0.04	0.01 ± 0.04	0.673	0.32 ± 0.07	0.36 ± 0.09	0.04 ± 0.45	0.001	0.03 ± 0.08	0.007	0.004

Abbreviations: HDL‐c, high‐density cholesterol; TB, theobromine.

^a^ Values are mean ± SD.

^b^ Based on paired samples *t*‐test.

^c^ Changes in the TB group − changes in the placebo group.

^d^ Independent samples *t*‐test.

^e^ Based on the ANCOVA test, adjusted for baseline values, changes in BMI and calorie intake.

### 3.3 Gene Expression Findings

The effects of TB on the gene expression of PPAR‐α and Sirt1 are shown in Table 6. Although a significant increase in the gene expression of PPAR‐α was observed after TB supplementation (*p* < 0.001), the results for Sirt1 reached no significant effect (*p* = 0.087). Moreover, the gene expressions of PPAR‐α according to $2^{-∆∆C_{t}}$ calculation were significantly increased in the TB group compared to the placebo, even after adjusting for confounders (*p* < 0.001). Our results also indicated that TB supplementation compared to the placebo had no significant effects on Sirt1 gene expression (Table 6).

**TABLE 6:** Comparison of gene expression of PPAR‐α and Sirt1 after 12 weeks according to intervention groups^a^ (data are presented as $2^{-∆∆C_{t}}$).

Variables	Placebo (*n* = 37)				TB (*n* = 35)				Difference^c^	*p*_between_ ^d^	*p*_between_ ^e^
	Baseline	Week 12	Change	*p* ^b^	Baseline	Week 12	Change	*p* ^b^			
Sirt1	1.631 ± 0.252	1.984 ± 0.259	0.353 ± 0.203	0.091	1.685 ± 0.26	2.332 ± 0.284	0.647 ± 0.367	0.087	0.294 ± 0.43	0.480	0.471
*PPAR‐α*	1.183 ± 0.130	1.516 ± 0.140	0.332 ± 0.178	0.07	1.46 ± 0.21	2.344 ± 0.17	0.884 ± 0.195	< 0.001	0.552 ± 0.20	0.043	0.004

Abbreviation: TB, theobromine.

^a^ Values are mean ± SD.

^b^ Based on paired samples *t*‐test.

^c^ Changes in the TB group − changes in the placebo group.

^d^ Independent samples *t*‐test.

^e^ Based on the ANCOVA test, adjusted for baseline values, changes in BMI and calorie intake.

## 4 Discussion

According to our current knowledge, this study is the first clinical trial to evaluate the effects of 450 mg/day TB supplementation in combination with a low‐calorie diet on serum levels of HDL‐c subgroups, the HDL‐c2/HDL‐c3 ratio, and the gene expression of PPAR‐α and Sirt1 in subjects with MetS. In the previous work (Sharifi‐Zahabi et al. 2023), we showed that TB significantly reduced WC by 0.8 cm compared to the placebo. Since both the TB and placebo groups followed an energy‐restricted diet, significant reductions of BMI and waist‐to‐hip ratio (WHR) were also observed in both arms. However, the difference between the two groups was statistically significant only for WC. Research has indicated that TB has positive effects on obesity and some elements of MetS, as demonstrated in both in vitro and in vivo studies (Wei et al. 2021; Jang et al. 2015). Additionally, TB diminished diet‐induced obesity in mice by promoting the browning of white adipose tissue (WAT) and stimulating brown adipose tissue (BAT) (Jang et al. 2020).

The current theory suggesting that TB may have anti‐obesity effects is mainly based on its stimulating impact on peroxisome proliferator‐activated receptor gamma coactivator (PGC)‐1α. This stimulation leads to the upregulation of PPAR‐α gene expression, which subsequently enhances β‐oxidation of fatty acids, reduces adipocyte hypertrophy, and improves inflammation in adipose tissue (Wei et al. 2021). In this study, the lack of a significant difference in body weight between groups may be associated with the maintenance of lean body mass resulting from TB consumption, as noted in a study by Wei et al. (2021). Earlier research indicated that weight reduction resulting from an energy‐restricted diet is attributed to decreases in both body fat mass and fat‐free mass (Willoughby et al. 2018; De Souza et al. 2012). However, weight loss when TB is incorporated into an energy‐restricted diet may stem from a reduction in body fat mass while preserving muscle mass. Nevertheless, we could not assess body composition due to the unavailability of body composition analysis techniques.

The results regarding HDL‐c and its subgroups showed a significant increase in the level of HDL‐c, HDL‐c2, and the HDL‐c2/HDL‐c3 ratio after receiving TB compared to the placebo. Moreover, TB supplementation led to an increase in the gene expression of PPAR‐α compared to the placebo. In contrast, in the placebo group, a significant reduction in HDL‐c was observed, likely due to weight loss associated with the low‐calorie diet. This finding indicates that TB supplementation may mitigate the reduction of HDL‐c following weight loss, highlighting its potential effect on lipid metabolism among subjects with MetS (Moradi et al. 2017). Low levels of HDL‐c are known as an independent risk factor for CVDs. Therefore, increasing the level of HDL‐c is considered a therapeutic strategy in CVDs management (Cho et al. 2020). In two previous studies, the effect of TB on HDL‐c yielded mixed results. In a study on healthy subjects, daily consumption of 850 mg/day TB for 4 weeks led to a significant increase in HDL‐c (6.2 mg/dL) compared to the placebo (Neufingerl et al. 2013), while in another one, intake of 500 mg/day TB by subjects with low baseline HDL‐c for 4 weeks did not result in a significant increase in HDL‐c (1.16 mg/dL) compared to the placebo (Smolders et al. 2018). The short duration of Smolders's study might be a reason for the nonsignificant results. Hence, our previous study with a longer duration revealed that TB intake had favorable effects on the LDL‐c/HDL‐c ratio, TG/HDL‐c ratio, TC/HDL‐c ratio, and serum levels of HDL‐c in overweight and obese subjects with MetS (Sharifi‐Zahabi et al. 2023).

Likewise, in this study, we found that TB supplementation could increase the serum levels of HDL‐c2 and the HDL‐c2/HDL‐c3 ratio, and decrease HDL‐c3 compared to the placebo. Therefore, the increased level of HDL‐c might be attributed to a rise in the HDL‐c2 subfraction. However, the magnitude of the increase in HDL‐c (1.58 mg/dL) is less than the reported MCID (minimal clinically important difference) for this lipoprotein (3.8 mg/dL) (Goldenberg et al. 2021). Therefore, the increases in HDL‐c and HDL‐c2 following TB supplementation, although statistically significant, are not clinically significant. Given the findings of the two previous studies (Smolders et al. 2018; Neufingerl et al. 2013), this nonclinically significant change in HDL‐c may be due to the health status of individuals who participated in this study and the low dose of TB supplementation. However, it should be noted that the dosage of TB used in the current study was selected based on previous studies (Smolders et al. 2018; Baggott et al. 2013) showing that TB intake at dietary relevant doses (250–500 mg/day) had fewer side effects.

Lagos et al. (2009) reported that lower HDL‐c levels in patients with MetS were due to a decrease in both large (HDL‐c2) and small (HDL‐c3) subfractions. Regarding HDL‐c3, a significant decrease was observed in both the placebo and TB groups. This decrease may be due to the effects of the low‐calorie diet on body weight, which often impacts lipid metabolism (Moradi et al. 2017), shifting the reduction in HDL‐c subfractions toward HDL‐c3. However, compared to the placebo, TB had no significant effect on HDL‐c3. Given the magnitude of the HDL‐c increase in this study, which was not large enough to reach clinical significance, the nonsignificant reduction effect of TB on HDL‐c3 compared to the placebo might be related to the health status of the study participants or the low dose of TB supplementation. Small, dense HDL‐c3 has high capacities to uptake cellular cholesterol, hinder the expression of cellular adhesion molecules, and prevent LDL oxidation compared to HDL‐c2 (de Souza et al. 2008). In a cohort study, Martin et al. (2015) showed that low levels of HDL‐c3 were associated with long‐term adverse clinical outcomes. It has been reported that the potent antioxidative properties of HDL‐c3 are impaired in MetS. Additionally, an increase in the number of MetS components is associated with a higher percentage of HDL‐c3 and a lower percentage of HDL‐c2 subfractions, which leads to a decrease in the HDL‐c2/HDL‐c3 ratio (Moriyama et al. 2014; Nikolic et al. 2013). Therefore, considering the aforementioned controversies regarding HDL‐c subfractions in MetS, it seems that assessing the HDL‐c2/HDL‐c3 ratio provides much more valuable information. However, only limited information is currently available regarding the association of the HDL‐c2/HDL‐c3 ratio, insulin resistance, related adipocytokines, and MetS. In this study, the protective role of HDL‐c against CVDs can be attributed to the altered HDL‐c functionality parameters like an increase in large particles (HDL‐c2) and the HDL‐c2/HDL‐c3 ratio. Indeed, a positive association between HDL‐c and the HDL‐c2/HDL‐c3 ratio and a negative link between the HDL‐c2/HDL‐c3 ratio and HOMA‐IR (Homeostatic Model Assessment for Insulin Resistance), BMI, WC, and TG has been reported (Moriyama et al. 2014). Therefore, the observed increase in the HDL‐c2/HDL‐c3 ratio in this study might be associated with reduced MetS risk factors. Insulin resistance is associated with several metabolic changes, including increased hepatic gluconeogenesis and glucose output, increased free fatty acid flow, hypertriglyceridemia, and decreased plasma HDL‐c levels (Lee et al. 2022; Muth et al. 2010). The low level of HDL‐c in subjects with insulin resistance is mainly due to the decrease in the level of HDL‐c2 and to a lesser extent HDL‐c3 (Muth et al. 2010). In line with these findings, the results of the current study also showed that the increase in HDL‐c2 level was associated with an increase in HDL‐c. The metabolism of HDL‐c is relatively complex, and a number of biological factors such as the enhancement in the synthesis of apolipoprotein A‐1, an increase in ABCA1 (adenosine triphosphate‐binding cassette transporter A1) mediated cholesterol efflux, and a decrease in the activity of the enzyme cholesteryl ester transfer protein (CETP) are known to affect serum levels of HDL‐c (Botta et al. 2018). Sirt1 and PPAR‐α play crucial roles in the regulation of metabolism, inflammation, and cellular responses in WBCs, and their expression can indeed be tissue specific, particularly concerning lipid metabolism (Felicidade et al. 2015; Li et al. 2024; Costa et al. 2021; Zeng et al. 2018; Song et al. 2011). The expression patterns of Sirt1 and PPAR‐α in WBCs often reflect those in lipid‐rich tissues. For example, during metabolic disorders or inflammation, both Sirt1 and PPAR‐α may be upregulated in WBCs, reflecting changes in lipid metabolism in other tissues (Costa et al. 2021; Song et al. 2011; Xu et al. 2024; Zamaninour et al. 2017; Ameer et al. 2017).

Although the exact mechanism for the effect of TB on HDL‐c and its subfraction is not fully understood, activation of PPAR‐α by the upstream gene, Sirt1, might be a suggested mechanism (Wei et al. 2021; Papadimitriou et al. 2015). In fact, it has been reported that PPAR‐α activation can lead to a decrease in TG synthesis and an increase in HDL‐c through increasing the expression of both ABCA1 and ApoA1 (apolipoprotein A1) (Matsuo 2022). Consequently, an increase in the HDL‐c2/HDL‐c3 ratio might indicate the increased cholesterol efflux to HDL‐c2 via ABCA1 and ABCG1 (adenosine triphosphate‐binding cassette transporter G1) activated by PPAR‐α (Matsuo 2022). Our results regarding Sirt1 showed no significant effect of TB. Till now, only one animal study, conducted on diabetic rats, has assessed the effect of TB on Sirt1 activity. In this study, Papadimitriou et al. (2015) revealed that administration of 5 mg/kg/day TB for 12 weeks could activate Sirt1 activity in the kidney and prevent diabetic nephropathy. Although a calorie restriction of 300–500 cal/day was prescribed for the study participants, analysis of dietary intakes revealed a calorie deficit of 200 kcal/day, approximately 10% of their total calorie intake. Therefore, our nonsignificant results for Sirt1 might be due to a low dose of TB supplementation and the low amount of calorie restriction, as the result of a study indicated that a 25% reduction in calorie intake is required to increase Sirt1 gene expression (Yu et al. 2018). Additionally, our findings indicate that the effect of TB on the PPAR‐α gene expression might be exerted through multiple pathways independent of Sirt1. These include direct activation via free fatty acids (Wei et al. 2021), signaling through AMPK (adenosine monophosphate‐activated protein kinase), interactions with coactivators of nuclear receptor (PGC‐1α) (Jang et al. 2018; Tuğal Aslan and Göktaş 2025), modulation by inflammatory, and responses to oxidative stress (Sugimoto et al. 2019). These pathways collectively enhance PPAR‐α's role in lipid metabolism and energy homeostasis (Wei et al. 2021; Todisco et al. 2022; Watt et al. 2004). However, future studies are required to confirm these findings.

The strengths of the current study include the larger sample size and longer duration compared to the previous studies, low dropout rates, prescription of a low‐calorie diet for each participant, high rate of compliance with interventions, and randomization of participants based on BMI categories for partially controlling the effect of BMI. However, self‐recording of dietary intakes and PA and lack of measurement of TB plasma concentration can be considered as limitations for the present study. Additionally, we did not evaluate the other parameters of HDL‐c functionality (CETP activity, ApoA1, etc.) due to financial constraints.

## 5 Conclusion

The results of the current study revealed that TB supplementation led to a statistically significant increase in serum levels of HDL‐c, HDL‐c2, the HDL‐c2/HDL‐c3 ratio, and the gene expression of PPAR‐α. However, further studies are needed to understand the possible underlying mechanisms and to assess other parameters of HDL‐c functionality.

## Author Contributions

**Elham Sharifi‐Zahabi:** conceptualization (equal), writing – original draft (equal), writing – review and editing (equal). **Zahra Dastafkan:** data curation (equal). **Amirhossein Asadi:** writing – original draft (equal). **Fatemeh Sadat Hosseini‐Baharanchi:** software (equal). **Nayebali Rezvani:** data curation (equal). **Saba Yari:** data curation (equal). **Farzad Shidfar:** conceptualization (equal), supervision (equal), writing – original draft (equal), writing – review and editing (equal).

## Ethics Statement

The ethics committee of Iran University of Medical Sciences, Tehran, Iran (IR.IUMS.REC.1400.761) approved the study protocol.

## Consent

Informed consent was obtained from all participants included in the study.

## Conflicts of Interest

The authors declare no conflicts of interest.

## Data Availability

The datasets generated and analyzed during the current study are available from the corresponding author upon reasonable request.
